# Trajectories of Prescription Drug Misuse Among US Adults From Ages 18 to 50 Years

**DOI:** 10.1001/jamanetworkopen.2021.41995

**Published:** 2022-01-04

**Authors:** Sean Esteban McCabe, John E. Schulenberg, Ty S. Schepis, Rebecca J. Evans-Polce, Timothy E. Wilens, Vita V. McCabe, Philip T. Veliz

**Affiliations:** 1 Center for the Study of Drugs, Alcohol, Smoking and Health, University of Michigan, Ann Arbor; 2 Institute for Social Research, University of Michigan, Ann Arbor; 3 Institute for Research on Women and Gender, University of Michigan, Ann Arbor; 4 Institute for Healthcare Policy and Innovation, University of Michigan, Ann Arbor; 5 Institute for Social Research, Department of Psychology, University of Michigan, Ann Arbor; 6 Department of Psychology, Texas State University, San Marcos; 7 Department of Psychiatry, Massachusetts General Hospital and Harvard Medical School, Boston; 8 Department of Surgery, University of Michigan, Ann Arbor; 9 Department of Psychiatry, University of Michigan, Ann Arbor

## Abstract

**Importance:**

US adults born from 1965 to 1996 had high exposure to controlled medications, yet little is known about how this exposure has affected them over time. Prescription drug misuse (PDM) has increased among adults in the past 2 decades, with related increases in emergency department visits, overdoses, and deaths.

**Objectives:**

To identify 32-year PDM trajectories involving opioids, stimulants, and sedatives or tranquilizers and to examine associations between these PDM trajectories and substance use disorder (SUD) symptoms in adulthood as well as between baseline characteristics and PDM trajectories.

**Design, Setting, and Participants:**

This cohort study included 11 cohorts of adolescents who were followed up longitudinally from age 18 years (study start, 1976-1986) to age 50 years (2008-2018) in the Monitoring the Future (MTF) study, which included a national multistage random sample of US 12th grade students. Baseline surveys (modal age 18) were self-administered in classrooms. Ten follow-ups were conducted by mail. Data analysis was conducted from December 2020 to October 2021.

**Main Outcomes and Measures:**

Sociodemographic variables were measured at baseline. PDM and SUD symptoms were measured at baseline and every follow-up. Latent profile analysis (LPA) was used to create PDM trajectory profiles. Associations between these PDM trajectories, SUD symptoms, and baseline sociodemographic characteristics were examined.

**Results:**

The sample of 26 575 individuals was 50.8% (95% CI, 50.2%-51.4%) female and 79.3% (95% CI, 78.8%-79.8%) White. The baseline response rate ranged from 77% to 84%, and the 32-year retention rate was 53%. In adjusting for attrition, 45.7% (95% CI, 44.9%-46.4%) of the respondents reported past-year PDM at least once during the 32-year reporting period. Among those who reported PDM, the prevalence of poly-PDM was 40.3% (95% CI, 39.3%-41.3%). Based on LPA, the number of class-specific PDM trajectories ranged from 4 (prescription opioids) to 6 (prescription stimulants). For the class-combined analyses, we identified 8 PDM trajectories consisting of early peak trajectories (eg, age 18 years), later peak trajectories (eg, age 40 years), and a high-risk trajectory (eg, high frequency PDM at multiple ages). All PDM trajectories were associated with increased odds of developing SUD symptoms in middle adulthood, especially the later peak and high-risk trajectories compared with early peak trajectories (eg, peak at age 40 years: adjusted odds ratio [aOR], 5.17; 95% CI, 3.97-6.73; high-risk: aOR, 12.41; 95% CI, 8.47-18.24). Baseline characteristics associated with a high-risk trajectory were binge drinking (aOR, 1.69; 95% CI, 1.13-2.54), cigarette smoking (aOR, 2.30; 95% CI, 1.60-3.29), and marijuana use (aOR, 3.78; 95% CI, 2.38-6.01). More recent cohorts (eg, 1985-1986) had a higher risk of belonging to later peak PDM trajectories (ages 40 and 45 years) than the 1976-1978 cohort (age 40 years peak: aOR, 2.49; 95% CI, 1.69-3.68).

**Conclusions and Relevance:**

In this cohort study, adults with later peak PDM trajectories were at increased risk of SUD symptoms in middle adulthood. These findings suggest the need to screen for PDM and SUD from adolescence through middle adulthood.

## Key Points

**Question:**

What are the trajectories of prescription drug misuse from adolescence to age 50 years, and which baseline characteristics and later substance-related problems are associated with these trajectories?

**Findings:**

In this cohort study of 26 575 individuals followed up from ages 18 to 50 years, nearly half of respondents reported prescription drug misuse. All prescription drug misuse trajectories had significantly increased odds of developing substance use disorder symptoms in adulthood, especially later peak trajectories, and baseline characteristics associated with prescription drug misuse trajectories included belonging to more recent cohorts, binge drinking, cigarette smoking, and using marijuana.

**Meaning:**

These findings suggest that clinicians should routinely screen for prescription drug misuse from adolescence throughout adulthood.

## Teaser

This cohort study identifies 32-year prescription drug misuse trajectories involving opioids, stimulants, and sedatives or tranquilizers and examines associations between these trajectories, substance use disorder (SUD) symptoms in adulthood, and baseline characteristics.

## Introduction

Individuals born between 1965 and 1996 represent more than 135 million adults in the United States who had high exposure to controlled medications, yet we know little about how this exposure was associated with long-term prescription drug misuse (PDM) in adulthood.^1,2^ PDM involving prescription opioids, stimulants, and sedatives or tranquilizers is most prevalent during young adulthood.^1,2^ In the past 2 decades, PDM has increased among adults, and there have been historic highs in PDM-related emergency department visits, overdoses, and deaths.^3,4,5^ Indeed, there were more than 96 000 past-year drug overdose US deaths.^3^ Therefore, it is important to identify high-risk PDM trajectories and early indicators associated with the development of substance-related problems later in adulthood.^6,7^

Most PDM research has been cross-sectional and lacked a developmental perspective that accounts for frequency of PDM and peak ages of frequent PDM.^7^ A few longitudinal studies have examined PDM trajectories in young adulthood, identifying experimental use as the most common type of PDM.^8,9^ Still, some individuals report persistent PDM into middle adulthood.^10^ Therefore, it is critical to extend the longitudinal study of PDM through middle adulthood, when PDM is less often experimental and more often associated with negative substance-related outcomes.^11^ The limited longitudinal studies examining PDM have focused on a single prescription drug class despite evidence that approximately 40% of PDM involves poly-PDM (misuse of 2 or more prescription medication classes).^10^ To address these gaps, we aimed to study 32-year PDM trajectories (ages 18-50 years) for several prescription drug classes, determine associated substance-related problems in middle adulthood, and examine baseline characteristics associated with PDM trajectories.

## Methods

### Data and Sample

The current study used panel data from the Monitoring the Future (MTF) study.^2,12^ Based on a 3-stage sampling procedure, MTF surveyed nationally representative samples of approximately 15 000 US high school seniors each year since 1975 using self-administered questionnaires. Parents received a waiver of informed consent, providing them a means to decline their child’s participation after receiving a complete description of the study. The analytic sample (26 575 participants) contained data from 11 cohorts of high school seniors (1976-1986) who were surveyed at modal age 18 years (baseline). The baseline response rates during the study period (1976-1986) ranged from 77% to 84%, with most nonresponse due to absence. The MTF panel oversampled 12th grade individuals who reported drug use, and weights were then used to approximate population estimates in the follow-ups. The mean weighted retention rate for the longitudinal samples from baseline (12th grade) to age 50 was 53%. To help correct for potential attrition bias and consistent with recent MTF panel analyses,^13,14^ we incorporated attrition weights to account for respondent characteristics associated with nonresponse at follow-up. The MTF study design, protocol, and sampling methods are described in greater detail elsewhere.^2,12^ This study meets the Strengthening the Reporting of Observational Studies in Epidemiology (STROBE) reporting guideline for cohort studies. Because the present study used deidentified data, it was deemed exempt by the University of Michigan institutional review board.

### Measures

Past-year PDM was measured at baseline and each follow-up with identical questions based on separate measures assessing past–12-month misuse of prescription opioids, stimulants, and sedatives or tranquilizers (“… taken any … on your own—that is, without a doctor telling you to take them?”). Respondents were provided a list of several generic and brand name examples for each of the prescription drug classes. The response scales for the questions ranged from 1, indicating no occasions, to 7, indicating 40 or more occasions. Each measure was treated as a continuous variable in the analyses to assess mean frequency. To assess combined PDM frequency including measures of opioids, stimulants, and sedatives or tranquilizers, the frequency of the highest prescription drug class misused was used as the indicator for PDM frequency at each specific wave (an overall measure of the highest frequency of PDM).

Substance use disorder (SUD) symptoms (measured at ages 35, 40, 45, and 50 years) were measured with several questions based on the *Diagnostic and Statistical Manual of Mental Disorders* (*DSM*) criteria for alcohol use disorder (AUD), cannabis use disorder (CUD), and other drug use disorder (ODUD). Fifteen items were used to characterize 8 of the 11 *DSM-5* criteria that define SUDs: (1) substance use resulting in a failure to fulfill major role obligations; (2) continued substance use when physically hazardous; (3) continued substance use despite persistent or recurrent interpersonal or social problems; (4) tolerance; (5) withdrawal; (6) persistent desire or unsuccessful efforts to cut down substance use; (7) health-related issue(s) due to substance use; and (8) craving. The criteria were summed to obtain an overall number of criteria endorsed. Although these measures of SUD symptoms do not yield a clinical diagnosis, the items we used are consistent with SUD measurements in other large-scale surveys^13,14,15^ and reflect *DSM-IV* and *DSM-5* AUD, CUD and ODUD symptoms.^16,17,18,19^ We followed recommended practice that meeting 2 or more criteria indicated any use disorder (mild, moderate, severe). This resulted in estimates closely resembling other national estimates for similar age groups.^20,21^

Sociodemographic variables and substance use behaviors at baseline included the following self-reported measures: sex, race and ethnicity, US Census geographic region, urbanicity based on metropolitan statistical area, parental education, college aspirations, average grade in high school, cohort year, 30-day cigarette use, 2-week binge drinking, and 30-day marijuana use. Race and ethnicity options were defined by the MTF study team and included Hispanic, non-Hispanic Black, non-Hispanic White, and other. Other was defined as American Indian, Asian, those who selected multiple races and ethnicities, and those with missing racial information. Previous research indicated that PDM varies by race and ethnicity,^1,2,7,10^ making it important to include race and ethnicity in these analyses. The following additional measures were included: educational attainment and marital status at age 50 years.

### Statistical Analysis

First, latent profile analysis (LPA) was used to create PDM trajectory profiles based on the number of past-year occasions that respondents misused each of the 3 prescription drug classes (opioids, stimulants, sedatives/tranquilizers), along with the composite PDM measures, during each specific follow-up; 4 separate LPAs were conducted. LPA is a statistical method that identifies subgroups of individuals based on multiple observed indicators (eg, frequency of PDM use across several time points). For the purposes of this analysis, we identified unique subgroups of respondents based on PDM frequency between age 18 and 50 years. For the analysis, only respondents who indicated past-year PDM at any wave were included in the LPA; respondents with no past-year PDM within each specific drug class across all waves were treated as a known class in subsequent analyses. The exploratory LPA (with no covariates) was conducted using Mplus version 8.0 (Muthén & Muthén). For each of the 4 LPAs, model fit was compared across 1 to 10 class solutions. Model fit was assessed using the Bayesian information criterion (BIC), Lo-Mendell-Rubin adjusted likelihood ratio test, and entropy measures. Lower BIC indicates better fit, and higher entropy indicates better separation of subgroups. Class membership was determined using a modal approach (a distinct separation of the sample based on assignment into each latent profile), which involved identifying and assigning individuals into the highest posterior-predicted probability of class membership for each of the respondents based on the best-fitting model.^22^ The resulting groups were then profiled and defined (groups were extracted and analyzed after the first step given the exploratory nature of the study).

Second, logistic regression models were fitted using the generalized estimating equations (GEE) methodology,^23,24^ with an autoregressive correlation structure (to account for the panel design) to assess the association between the trajectory groups and the past 5-year prevalence of SUD symptoms during the 15-year period in middle adulthood (when accounting for the key control variables). The results were similar for the GEE models when either an unstructured or exchangeable correlation structure was used.

Third, multinomial logistic regression was used to examine how several key sociodemographic characteristics and substance use behaviors at baseline were associated with each PDM trajectory; relative risk ratios (RRRs) and 95% CIs were reported using the no PDM trajectory as the reference category. For the analyses, all respondents were included when possible. The LPA estimated in Mplus used full information likelihood estimation to handle missing data. With respect to assessing the association between trajectories, SUD, and baseline characteristics, sample sizes varied across analyses due to responses with missing items. All descriptive and regression analyses were conducted using Stata 15.0 (StataCorp). Statistical significance was set at *P* < .05, and all tests were 2-tailed.

## Results

### Sample Characteristics

Table 1 provides the baseline sociodemographic characteristics. The sample was 50.8% (95% CI, 50.2%-51.4%) female. An estimated 45.7% (95% CI, 44.9%-46.4%) reported past-year PDM at least once during the 32-year period. Among those who reported PDM, 40.3% (95% CI, 39.3%-41.3%) reported poly-PDM (ie, past-year misuse of more than 1 prescription drug class in the same wave).

**Table 1.** Sample Characteristics^a^

Baseline characteristics	Participants, No. (%) (N = 26 575)
Sex	
Male	13 073 (49.2)
Female	13 496 (50.8)
Missing	6 (0.02)
Race and ethnicity^b^	
Black (non-Hispanic)	2846 (10.7)
Hispanic	946 (3.6)
White (non-Hispanic)	21 082 (79.3)
Other^c^	1701 (6.4)
GPA	
B− or higher	17 917 (69.8)
C+ or lower	7735 (30.2)
Missing	923 (3.5)
College plans	
No	11 314 (45.6)
Yes	13 522 (54.4)
Missing	1736 (6.5)
Parental education level	
Less than a college degree	16 458 (64.7)
College degree or higher	8964 (35.3)
Missing	1153 (4.3)
Urbanicity	
Urban	7072 (26.6)
Suburban	11 206 (42.2)
Rural	8297 (31.2)
US region	
Northeast	6441 (24.2)
Midwest	7882 (29.7)
South	7937 (29.9)
West	4315 (16.2)
Cohort year	
1976-1978	6993 (26.3)
1979-1981	7353 (27.7)
1982-1984	7301 (27.5)
1985-1986	4928 (18.5)
30-d Cigarette use	
No	15 761 (60.5)
Yes	10 295 (39.5)
Missing	519 (2.0)
2-wk Binge drinking	
No	13 098 (52.3)
Yes	11 952 (47.7)
Missing	1525 (5.7)
30-d Marijuana use	
No	14 733 (57.4)
Yes	10 947 (42.6)
Missing	895 (3.4)

Abbreviation: GPA, grade point average.

^a^ At baseline, 26 575 high school seniors were selected into the longitudinal sample between 1976 and 1986; 2748 respondents did not complete any follow-up between ages of 19 to 20 and 50 years. Unweighted sample sizes and percentages are provided.

^b^ Measures for race and ethnicity were self-reported and predefined within the survey. Race was assessed because of differences across behavioral and social outcomes.

^c^ Other race and ethnicity was defined as American Indian, Asian, those who selected multiple races and ethnicities, and those with missing race and ethnicity information.

### PDM Trajectories From Adolescence to Age 50 Years

The results of the LPA indicated a range of multiple class solutions that provided the best fit for opioids (4-class solution), stimulants (6-class solution), tranquilizer or sedatives (4-class solution), and the composite PDM measure (8-class solution) (eTables 1-4 in the Supplement). The Figure provides the trajectories for opioids, stimulants, tranquilizer/sedatives, and the composite PDM measure. A large portion of past-year PDM for each class of prescription drugs peaked at age 18 years. Each PDM class had distinct trajectories that peaked in either the mid-20s or middle adulthood (age 35 years and older); peaks in early and middle adulthood had greater PDM frequency compared with peaks at age 18 years. Prescription stimulants had multiple trajectories that peaked later in middle adulthood (ages 40, 45, and 50 years).

**Figure.**
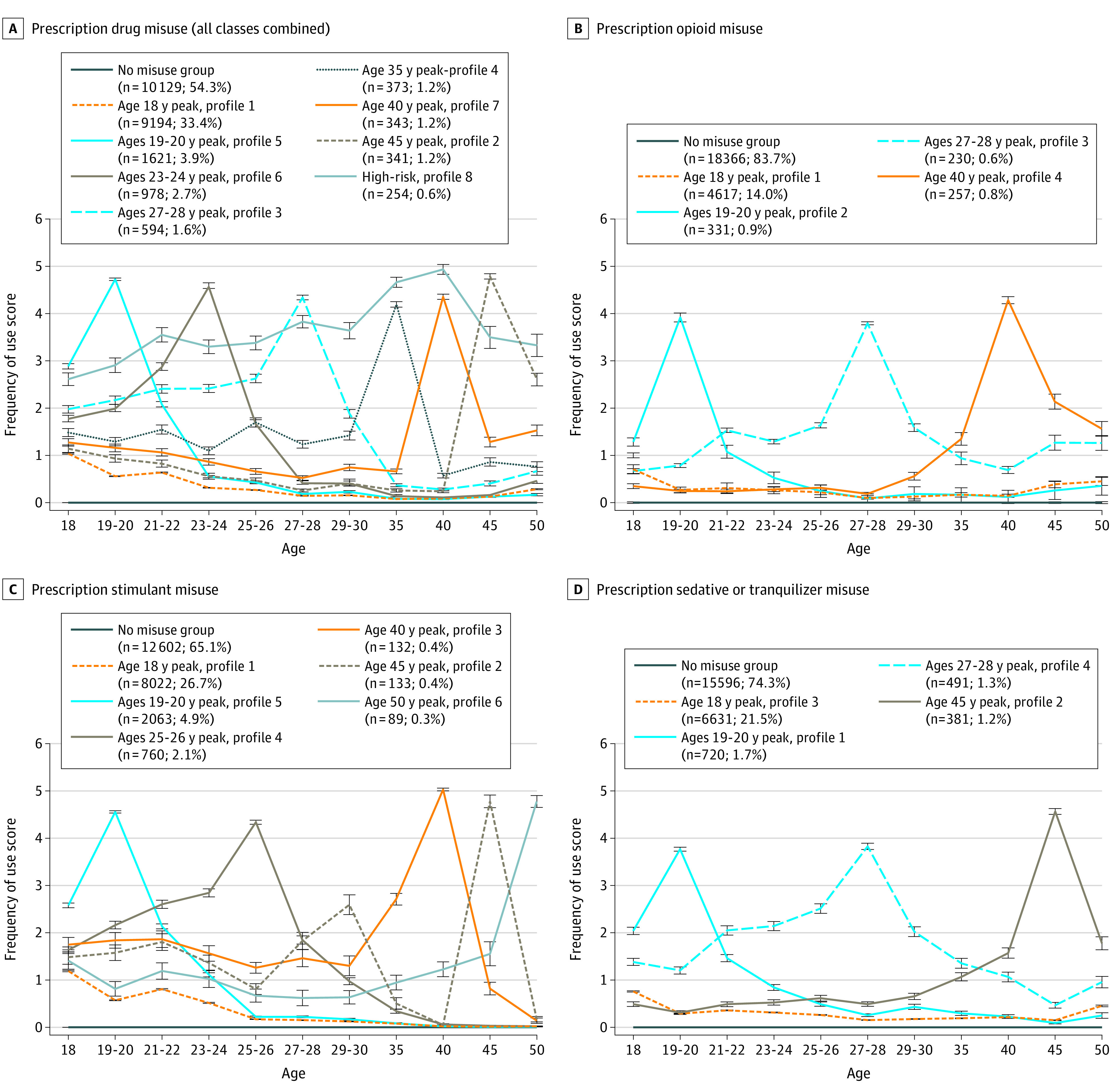
Latent Profiles of Past-Year Prescription Drug Misuse Between Ages 18 and 50 Years Frequency of use scores range from 0 (no use) to 6 (≥40 occasions). Error bars represent 95% CIs based on the standard error of the mean frequency score.

### PDM Trajectories and SUD Symptoms From Ages 35 to 50 Years

In Table 2, all PDM trajectories had significantly increased odds of 2 or more AUD, CUD, and/or ODUD symptoms from ages 35 to 50 years compared with the no PDM trajectory after controlling for baseline drug use covariates and time of survey collection in adulthood (ages 35, 40, 45, and 50 years). Moreover, trajectories that peaked in early adulthood (ages 23-24, 25-26, and 27-28 years) and middle adulthood (ages 35, 40, 45, and 50 years) across each class of PDM were associated with significantly increased odds of indicating 2 or more SUD symptoms compared with the age 18 years peak trajectory (eg, age 40 years peak: adjusted odds ratio [aOR], 5.17; 95% CI, 3.97-6.73). The high-risk trajectory was associated with much greater odds of at least 2 symptoms of any SUD (aOR, 12.41; 95% CI, 8.47-18.24). The corresponding prevalence estimates of 2 or more SUD symptoms from ages 35 to 50 years for the no PDM trajectory (26.0% [95% CI, 24.8%-27.1%]) was much lower than the early peak PDM trajectories at ages 18 or 19 to 20 years (48.0% [95% CI, 46.5%-49.4%] and 60.1% [95% CI, 56.3%-63.7%], respectively), the early adulthood peak at ages 23 to 24 or 27 to 28 years (66.8% [95% CI, 62.6%-70.6%] and 78.3% [95% CI, 73.6%-82.3%], respectively), the middle adulthood peak at ages 35, 40, or 45 years (79.2% [95% CI, 73.1%-84.2%], 71.8% [95% CI, 65.7%-77.2%], and 75.1% [95% CI, 68.8%-80.3%], respectively), and the high-risk trajectory (94.0% [95% CI, 86.2%-97.7%]).

**Table 2.** Associations Between Prescription Drug Misuse Trajectories and SUD Symptoms During Adulthood^a^

Trajectory	aOR (95% CI)			
	≥2 AUD symptoms (n = 11 435)	≥2 CUD symptoms (n = 11 363)	≥2 ODUD symptoms (n = 11 088)	≥2 Symptoms for any SUD (n = 11 582)
**Prescription drug misuse trajectories**				
No prescription drug misuse trajectory	1 [Reference]	1 [Reference]	1 [Reference]	1 [Reference]
Peak trajectory				
Age 18 y	1.84 (1.66-2.04)	4.53 (3.38-6.09)	5.29 (3.45-8.13)	1.96 (1.77-2.17)
Age 19-20 y	2.17 (1.82-2.59)	6.74 (4.61-9.85)	6.66 (2.78-7.38)	2.36 (1.98-2.80)
Age 23-24 y	2.89 (2.39-3.49)	9.73 (6.62-14.34)	10.58 (3.98-11.13)	3.20 (2.65-3.86)
Age 27-28 y	3.31 (2.65-4.13)	11.91 (7.81-18.33)	19.10 (2.51-72.69)	4.35 (3.48-5.42)
Age 35 y	3.82 (2.88-5.08)	18.63 (12.11-28.67)	45.34 (11.61-313.98)	5.50 (4.14-7.32)
Age 40 y	4.01 (3.08-5.20)	12.24 (8.11-18.39)	55.32 (6.46-174.22)	5.17 (3.97-6.73)
Age 45 y	4.42 (3.38-5.78)	15.22 (9.31-24.87)	42.73 (7.37-223.89)	6.05 (4.61-7.95)
High risk across multiple ages trajectory	4.98 (3.54-7.00)	25.73 (15.88-41.49)	252.03 (33.66-430.68)	12.41 (8.47-18.24)
Time – time-varying (0, age 35 y, to 3, age 50 y)	0.82 (0.80-0.84)	0.67 (0.64-0.70)	0.76 (0.71-0.81)	0.83 (0.81-0.85)
**Prescription opioid misuse trajectories**				
No prescription opioid misuse trajectory	1 [Reference]	1 [Reference]	1 [Reference]	1 [Reference]
Peak trajectory				
Age 18 y	1.92 (1.74-2.12)	3.61 (3.03-4.31)	4.09 (3.29-5.09)	2.14 (1.94-2.36)
Age 19-20 y	1.56 (1.12-2.18)	3.74 (2.47-5.70)	5.62 (3.49-9.05)	2.12 (1.53-2.94)
Age 27-28 y	2.53 (1.76-3.65)	4.91 (2.89-8.36)	12.93 (7.85-21.37)	3.44 (2.31-5.10)
Age 40 y	4.92 (3.53-6.87)	6.73 (4.61-9.81)	38.75 (27.41-54.88)	6.64 (4.75-9.30)
Time – time varying (0, age 35 y, to 3, age 50 y)	0.82 (0.80-0.84)	0.66 (0.63-0.70)	0.77 (0.72-0.82)	0.83 (0.81-0.85)
**Prescription stimulant misuse trajectories**				
No prescription stimulant misuse trajectory	1 [Reference]	1 [Reference]	1 [Reference]	1 [Reference]
Peak trajectory				
Age 18 y	1.61 (1.45-1.78)	3.35 (2.69-4.18)	3.35 (2.53-4.45)	1.75 (1.59-1.94)
Age 19-20 y	1.96 (1.69-2.27)	5.03 (3.75-6.74)	4.33 (3.08-6.09)	2.25 (1.93-2.61)
Age 25-26 y	3.11 (2.53-3.82)	7.61 (5.42-10.57)	10.93 (7.74-15.49)	3.90 (3.17-4.81)
Age 40 y	3.04 (2.01-4.59)	12.68 (7.80-20.86)	60.33 (35.68-101.61)	5.96 (4.00-8.90)
Age 45 y	2.95 (1.74-5.02)	6.17 (2.48-15.34)	18.94 (8.54-42.04)	3.84 (2.22-6.64)
Age 50 y	2.68 (1.65-4.35)	11.81 (5.87-23.73)	29.37 (18.06-47.72)	4.77 (2.96-7.70)
Time – time varying (0, age 35 y, to 3, age 50 y)	0.83 (0.81-0.88)	0.67 (0.64-0.70)	0.78 (0.74-0.83)	0.83 (0.80-0.85)
**Prescription sedative or tranquilizer misuse trajectories**				
No sedative or tranquilizer misuse trajectory	1 [Reference]	1 [Reference]	1 [Reference]	1 [Reference]
Peak trajectory				
Age 18 y	1.80 (1.64-1.98)	2.89 (2.39-3.48)	4.63 (3.68-5.83)	1.98 (1.80-2.17)
Age 19-20 y	1.64 (1.31-2.05)	3.24 (2.32-4.52)	4.30 (2.99-6.19)	1.83 (1.46-2.29)
Age 27-28 y	2.52 (1.95-3.25)	5.44 (3.77-7.85)	14.12 (9.57-20.75)	3.27 (2.51-4.25)
Age 45 y	3.19 (2.49-4.09)	7.50 (5.10-11.03)	21.08 (14.72-30.19)	4.33 (3.34-5.60)
Time – time varying (0, age 35 y, to 3, age 50 y)	0.82 (0.81-0.84)	0.67 (0.64-0.70)	0.78 (0.74-0.83)	0.83 (0.81-0.85)

Abbreviations: aOR, adjusted odds ratio; AUD, alcohol use disorder; CUD, cannabis use disorder; ODUD, other drug use disorder; SUD, substance use disorder.

^a^ Unweighted samples sizes are provided. All estimates provided use weights to adjust for attrition at age 50 years. Models control for the following time invariant baseline variables: sex, race and ethnicity, parental level of education, urbanicity, US region, cohort year, average grade point average in their senior year, aspirations to attend college, past 30-day cigarette use, past 2-week binge drinking, and past 30-day marijuana use; models also controlled for college attainment and marital status at age 50 years. Measures for race and ethnicity were self-reported and predefined within the survey. Race was assessed because of differences across behavioral and social outcomes. Other race and ethnicity was defined as American Indian, Asian, those who selected multiple races and ethnicities, and those with missing race and ethnicity information.

### Baseline Characteristics Associated With PDM Trajectory Membership

As shown in Table 3 and eTables 5, 6, and 7 in the Supplement, the baseline measures that were significantly associated with PDM trajectory membership included: cigarette use (aOR, 2.30; 95% CI, 1.60-3.29), binge drinking (aOR, 1.69; 95% CI, 1.13-2.54), and marijuana use (aOR, 3.78; 95% CI, 2.38-6.01). Respondents who identified as Black (non-Hispanic) had lower expected risk of being in any of the PDM trajectories. More recent cohorts (1985-1986) had a higher risk of belonging to later peak PDM trajectories (ages 40 and 45 years) and lower risk of belonging to early peak PDM trajectories (ages 18 and 19-20 years) than the 1976-1978 cohort (age 40 years peak: aOR, 2.49; 95% CI, 1.69-3.68; age 19-20 years peak: aOR, 0.58; 95% CI, 0.44-0.76). Moreover, medical use of prescription drugs at age 18 years was associated with greater risk of belonging to young adulthood–peak PDM trajectories. PDM at age 18 years was associated with greater risk of belonging to most PDM trajectories.

**Table 3.** Multinomial Logistic Regression Assessing Characteristics Associated With Membership in Prescription Drug Misuse Trajectories^a^

Baseline characteristic	aOR (95% CI)							
	Peak trajectory							High-risk trajectory
	Age 18 y	Ages 19-20 y	Ages 23-24 y	Ages 27-28 y	Age 35 y	Age 40 y	Age 45 y	
Sex								
Male	1 [Reference]	1 [Reference]	1 [Reference]	1 [Reference]	1 [Reference]	1 [Reference]	1 [Reference]	1 [Reference]
Female	1.17 (1.09-1.25)	1.26 (1.10-1.45)	0.91 (0.76-1.08)	1.19 (0.95-1.49)	1.13 (0.87-1.47)	0.93 (0.70-1.25)	1.36 (1.01-1.83)	0.86 (0.62-1.18)
Race and ethnicity^b^								
White non-Hispanic	1 [Reference]	1 [Reference]	1 [Reference]	1 [Reference]	1 [Reference]	1 [Reference]	1 [Reference]	1 [Reference]
Black non-Hispanic	0.59 (0.51-0.68)	0.39 (0.25-0.60)	0.42 (0.26-0.67)	0.19 (0.08-0.46)	0.16 (0.05-0.54)	0.44 (0.23-0.85)	0.58 (0.29-1.14)	0.28 (0.09-0.82)
Hispanic	0.86 (0.70-1.06)	0.90 (0.55-1.45)	0.79 (0.38-1.63)	0.52 (0.22-1.25)	0.34 (0.13-0.91)	0.72 (0.25-2.06)	1.19 (0.58-2.44)	0.92 (0.35-2.38)
Other^c^	0.77 (0.66-0.90)	1.05 (0.73-1.50)	0.96 (0.64-1.43)	1.00 (0.59-1.68)	0.95 (0.54-1.70)	0.70 (0.35-1.40)	1.01 (0.54-1.90)	0.41 (0.20-0.83)
Parental education								
Less than a BA	1 [Reference]	1 [Reference]	1 [Reference]	1 [Reference]	1 [Reference]	1 [Reference]	1 [Reference]	1 [Reference]
BA or higher	1.11 (1.02-1.19)	1.17 (1.01-1.36)	1.06 (0.88-1.28)	0.82 (0.64-1.05)	0.99 (0.74-1.31)	1.09 (0.79-1.50)	0.93 (0.69-1.24)	0.78 (0.55-1.09)
GPA								
B− or higher	1 [Reference]	1 [Reference]	1 [Reference]	1 [Reference]	1 [Reference]	1 [Reference]	1 [Reference]	1 [Reference]
C+ or lower	1.07 (0.99-1.16)	0.97 (.822-1.13)	0.92 (0.76-1.10)	1.31 (1.04-1.65)	1.38 (1.01-1.88)	0.90 (0.63-1.26)	1.11 (0.81-1.53)	0.92 (0.62-1.34)
College plans								
No	1 [Reference]	1 [Reference]	1 [Reference]	1 [Reference]	1 [Reference]	1 [Reference]	1 [Reference]	1 [Reference]
Yes	1.05 (0.98-1.14)	0.94 (0.81-1.09)	0.79 (0.66-0.96)	0.88 (0.69-1.12)	0.96 (0.71-1.30)	0.88 (0.65-1.19)	0.95 (0.70-1.28)	0.89 (0.63-1.25)
Region								
Northeast	1 [Reference]	1 [Reference]	1 [Reference]	1 [Reference]	1 [Reference]	1 [Reference]	1 [Reference]	1 [Reference]
Midwest	0.95 (0.87-1.04)	1.12 (0.95-1.34)	1.46 (1.16-1.83)	1.45 (1.06-1.97)	1.05 (0.69-1.59)	0.96 (0.65-1.40)	1.08 (0.75-1.56)	0.89 (0.58-1.36)
South	1.01 (0.92-1.11)	1.07 (0.88-1.31)	1.40 (1.09-1.80)	1.54 (1.12-2.11)	1.39 (0.91-2.14)	1.51 (1.02-2.23)	0.96 (0.64-1.44)	0.97 (0.60-1.56)
West	1.05 (0.95-1.18)	1.21 (0.97-1.52)	1.30 (0.97-1.77)	1.73 (1.21-2.49)	1.89 (1.24-2.88)	1.74 (1.10-2.75)	1.16 (0.76-1.77)	1.63 (1.04-2.54)
Urbanicity								
Urban	1 [Reference]	1 [Reference]	1 [Reference]	1 [Reference]	1 [Reference]	1 [Reference]	1 [Reference]	1 [Reference]
Suburban	1.08 (1.00-1.19)	0.93 (0.78-1.10)	1.11 (0.90-1.38)	1.17 (0.88-1.56)	1.43 (1.00-2.03)	1.04 (0.72-1.51)	1.09 (0.75-1.58)	1.62 (1.08-2.45)
Rural	1.10 (1.00-1.21)	1.02 (0.85-1.23)	1.01 (0.80-1.29)	1.08 (0.81-1.44)	1.41 (0.97-2.04)	0.84 (0.57-1.23)	0.95 (0.64-1.41)	1.58 (1.03-2.43)
Cohort year								
1976-1978	1 [Reference]	1 [Reference]	1 [Reference]	1 [Reference]	1 [Reference]	1 [Reference]	1 [Reference]	1 [Reference]
1979-1981	1.04 (0.95-1.14)	1.60 (1.36-1.90)	0.53 (0.44-0.64)	0.45 (0.95-1.14)	0.61 (0.43-0.87)	0.93 (0.62-1.40)	1.87 (1.25-2.80)	0.78 (0.54-1.11)
1982-1984	1.02 (0.93-1.12)	0.93 (0.78-1.12)	0.30 (0.246-0.39)	0.36 (0.25-0.39)	0.79 (0.56-1.11)	1.55 (1.05-2.30)	1.60 (1.04-2.47)	0.73 (0.48-1.12)
1985-1986	0.87 (0.78-0.96)	0.58 (0.44-0.76)	0.13 (0.09-0.20)	0.32 (0.24-0.39)	0.91 (0.61-1.33)	2.49 (1.69-3.68)	1.73 (1.10-2.73)	0.44 (0.25-0.77)
Cigarette use								
No	1 [Reference]	1 [Reference]	1 [Reference]	1 [Reference]	1 [Reference]	1 [Reference]	1 [Reference]	1 [Reference]
Yes	1.26 (1.16-1.37)	1.80 (1.53-2.13)	1.37 (1.12-1.68)	1.40 (1.09-1.80)	1.92 (1.40-2.64)	1.29 (0.89-1.89)	1.17 (0.88-1.56)	2.30 (1.60-3.29)
Binge drinking								
No	1 [Reference]	1 [Reference]	1 [Reference]	1 [Reference]	1 [Reference]	1 [Reference]	1 [Reference]	1 [Reference]
Yes	1.37 (1.26-1.48)	2.14 (1.79-2.55)	1.65 (1.33-2.04)	1.75 (1.31-2.33)	1.34 (0.96-1.87)	1.77 (1.26-2.50)	1.28 (0.92-1.79)	1.69 (1.13-2.54)
Marijuana use								
No	1 [Reference]	1 [Reference]	1 [Reference]	1 [Reference]	1 [Reference]	1 [Reference]	1 [Reference]	1 [Reference]
Yes	1.91 (1.75-2.08)	4.22 (3.44-5.17)	3.06 (2.44-3.84)	2.73 (2.03-3.67)	1.74 (1.23-2.48)	1.43 (1.01-2.03)	1.46 (1.03-2.05)	3.78 (2.38-6.01)
Lifetime medical and nonmedical use^d^								
Never	1 [Reference]	1 [Reference]	1 [Reference]	1 [Reference]	1 [Reference]	1 [Reference]	1 [Reference]	1 [Reference]
Medical only	1.22 (0.95-1.56)	0.95 (0.34-2.66)	2.97 (1.38-6.37)	2.11 (0.80-5.58)	0.84 (0.22-3.19)	2.09 (0.76-5.78)	1.47 (0.68-3.21)	NA^e^
Nonmedical only	5.29 (4.10-6.82)	4.98 (2.60-9.52)	3.79 (1.72-8.35)	2.15 (0.93-5.00)	2.08 (0.81-5.38)	3.57 (1.31-9.71)	1.23 (0.50-3.08)	7.50 (1.86-30.24)
Both	4.51 (3.55-5.73)	5.34 (2.79-10.21)	2.97 (1.43-6.17)	4.21 (2.00-8.88)	3.00 (1.34-6.72)	2.42 (0.96-6.18)	0.96 (0.40-2.28)	7.82 (2.35-26.03)

Abbreviations: aOR, adjusted odds ratio; GPA, grade point average; NA, not applicable.

^a^ The overall sample size was 19 915, and individual trajectories ranged from 254 participants (high risk) to 9194 participants (age 18 years peak). All estimates provided use weights to adjust for attrition at age 50 years.

^b^ Measures for race and ethnicity were self-reported and predefined within the survey. Race was assessed because of differences across behavioral and social outcomes.

^c^ Other race and ethnicity was defined as American Indian, Asian, those who selected multiple races and ethnicities, and those with missing race and ethnicity information.

^d^ Seperate analyses with lifetime medical and nonmedical use at age 18 years could only include one-sixth of the sample due to form-specific questions given to 3685 respondents at baseline.

^e^ No respondents who were in this trajectory group indicated medical only use at baseline.

## Discussion

To our knowledge, this is the first longitudinal US national multicohort study to examine PDM trajectories involving prescription opioids, stimulants, and sedatives or tranquilizers from ages 18 to 50 years. The LPA confirmed prior studies that have found unique groups of individuals that have peak PDM in young adulthood^8,9,10^; moreover, the present study identified additional PDM trajectories that were characterized by frequent PDM at later ages in adulthood, peaking between ages 40 and 50 years. Although each of these later peak trajectories included less than 3% of the sample, the individuals within the later peak PDM trajectories had significantly higher risk of SUD in middle adulthood compared with trajectories with earlier peaks. Individuals in later peak PDM trajectories were more common in recent cohorts than in past cohorts, suggesting the middle adulthood peaks are now more of a public health concern.

Our findings extend what is known about the development of PDM trajectories from adolescence to middle adulthood in 3 ways. First, we found evidence that 45.7% of the sample reported any past-year PDM at some point over a 32-year period. The majority who reported PDM peaked at age 18 years, followed by trajectories that peaked at ages 19 to 20 and 23 to 24 years; the remaining PDM trajectories, constituting approximately 1 in every 8 individuals who reported PDM, peaked between ages 27 to 28 and 45 years, highlighting distinct developmental courses where the most frequent PDM occurred later in life. Most individuals who were classified as belonging to the peak PDM at age 40 or later trajectories had symptoms consistent with an SUD in adulthood; in addition, these PDM trajectories showed relatively sharp increases to their peaks. These peak PDM ages coincide with the highest rates of prescription opioid-involved overdose, which are among individuals aged 45 to 54 years, followed closely by those aged 35 to 44 years.^5^ Accounting for the age when PDM occurs, as well as frequency of misuse, is critical when assessing an individual’s risk of developing substance-related problems.

Second, all PDM trajectories were at increased risk of SUD symptoms in middle adulthood, regardless of peak PDM age when compared with individuals who never engaged in PDM. Notably, PDM trajectories that peaked at age 18 years, with decreased PDM thereafter, had a significantly lower risk of SUD compared with PDM trajectories with later peaks. Thus, for clinicians, PDM at any age should be viewed as a signal for subsequent substance-related problems and included in screening from adolescence through middle adulthood.^3^

Third, we identified several baseline and sociodemographic characteristics associated with PDM trajectory membership. The most robust risk factors underscored the role of polysubstance use; binge drinking, cigarette smoking, and marijuana use at age 18 years were all associated with increased odds of belonging to a PDM trajectory group. We also found that Black (non-Hispanic) adolescents and adults had lower risk of being in a PDM trajectory group. Recent cohorts had higher risk of belonging to PDM trajectories that peaked at a later age relative to past cohorts, suggesting the middle-age peaks are more common in recent years. Medical use of a prescription drug (without a history of misuse) at age 18 years was associated with membership in peak PDM trajectories in young adulthood but was not associated with membership in PDM trajectories in middle adulthood. In contrast, a history of medical use and misuse of prescription drugs at age 18 was associated with membership in most PDM trajectories. Taken together, a comprehensive screening for substance use history can alert clinicians to subsequent risk for PDM and substance-related problems at specific ages and enhance precision medicine efforts.^25^

Previous work examining PDM trajectories from ages 18 to 35 years identified peaks at ages 18, 19 to 20, 23 to 24, and 27 to 28 using MTF data, while the present study evaluated individuals from ages 18 to 50 years.^10^ During the 32-year time frame, the present study found additional PDM peaks at later ages (eg, 40 years or older) for each class as well as the PDM trajectories that peaked in young adulthood shown in previous studies. The present study found that the PDM trajectories that peaked in young adulthood tended to decrease to low or no PDM by ages 40 to 50 years. In contrast, for the PDM trajectories that peaked in middle adulthood, PDM frequency sharply increased during ages 35 to 50 years and was associated with higher risk of middle adulthood SUDs. A recent study found that most older adults who engaged in prescription opioid misuse reported physical pain relief as their main motivation, while young adults were more likely to report non–pain relief motivations, such as “to get high.”^26^ The PDM trajectories that peaked in middle adulthood identified in the present study may be misusing prescription opioids, stimulants, and sedatives or tranquilizers for different reasons than the adolescent and young adulthood peak trajectories. Motivations like physical pain relief or anxiety later in adulthood require different interventions than the experimental use often reported earlier in life. Our study promotes a developmental assessment of PDM to reduce risky substance use. Based on the study findings, screening instruments are recommended that not only focus on SUD, but also include PDM (such as the National Institute on Drug Abuse Tobacco, Alcohol, Prescription Medication, and Other Substance Use Tool, Quick Screen).

### Strengths and Limitations

Several strengths and limitations should be considered while evaluating the implications of the present study. The major strengths include US national samples of multiple cohorts that were followed up prospectively over 11 longitudinal waves from ages 18 to 50 years using the same study design and measures. The major limitations are common to large-scale prospective survey research studying high-risk behaviors, including differential attrition and survey measure limitations. The MTF study did not include all of the *DSM-5* SUD criteria; the actual prevalence of *DSM-5* SUD was likely higher. There could be overreporting and underreporting of PDM due to respondents not knowing the prescription drug class they misused. The exclusion of individuals who did not complete high school and institutionalized adults and the higher attrition among those who used drugs more frequently could have provided an underrepresentation of the most severe PDM trajectories; however, this limitation is partly mitigated by accounting for differential attrition in the analyses. Finally, while LPA is a robust statistical method to uncover various subpopulations based on PDM trajectories, this method is limited given that it is sensitive to misclassification, as additional covariates are added into these types of models. It should also be noted that the current study only engaged in an exploratory LPA and was simply used to uncover unique groupings of individuals based on PDM trajectories that will need to be confirmed in future research.

## Conclusions

Medical use of controlled medications is most prevalent during middle and older adulthood.^1,11^ The high medication usage, limited screening, and lack of adequate monitoring practices have contributed to increases in PDM among older adults and historic highs in PDM-related emergency department visits, overdoses, and deaths.^3,4,5,27^ Indeed, the present study found evidence that approximately 46% of adults misused prescription medications at least once between the ages of 18 and 50 years. While substance use prevention during adolescence is a justified public health focus, clinicians, prescribers, and researchers must better understand long-term PDM trajectories to reduce PDM-related consequences in later adulthood. The findings of the present study indicate that some US adults do not report their most frequent misuse until later in life, which reinforces the importance of educating about and screening for PDM and SUD from adolescence through middle adulthood.

## Notes

Supplement. **eTable 1.** Estimated Latent Profile Analysis for Past-Year Prescription Drug Misuse **eTable 2.** Estimated Latent Profile Analysis for Past-Year Prescription Opioid Misuse **eTable 3.** Estimated Latent Profile Analysis for Past-Year Prescription Stimulant Misuse **eTable 4.** Estimated Latent Profile Analysis for Past-Year Prescription Sedative or Tranquilizer Misuse **eTable 5.** Multinomial Logistic Regression Assessing Characteristics Associated With Membership in Prescription Opioid Misuse Trajectories **eTable 6.** Multinomial Logistic Regression Assessing Characteristics Associated With Membership in Prescription Stimulant Misuse Trajectories **eTable 7.** Multinomial Logistic Regression Assessing Characteristics Associated With Membership in Prescription Sedative or Tranquilizer Misuse Trajectories
