# Supplementary material for: Trajectories of Prescription Drug Misuse Among US Adults From Ages 18 to 50 Years
Source: JAMA Netw Open. 2022 Jan 4;5(1):e2141995. doi: 10.1001/jamanetworkopen.2021.41995 (PMC8728613; doi:10.1001/jamanetworkopen.2021.41995)
Supplement: Supplement. — eTable 1. Estimated Latent Profile Analysis for Past-Year Prescription Drug Misuse eTable 2. Estimated Latent Profile Analysis for Past-Year Prescription Opioid Misuse eTable 3. Estimated Latent Profile Analysis for Past-Year Prescription Stimulant Misuse eTable 4. Estimated Latent Profile Analysis for Past-Year Prescription Sedative or Tranquilizer Misuse eTable 5. Multinomial Logistic Regression Assessing Characteristics Associated With Membership in Prescription Opioid Misuse Trajectories eTable 6. Multinomial Logistic Regression Assessing Characteristics Associated With Membership in Prescription Stimulant Misuse Trajectories eTable 7. Multinomial Logistic Regression Assessing Characteristics Associated With Membership in Prescription Sedative or Tranquilizer Misuse Trajectories [file jamanetwopen-e2141995-s001.pdf]

Supplemental Online Content

McCabe SE, Schulenberg JE, Schepis TS, et al. Trajectories of prescription drug misuse among US adults from ages 18 to 50 years. *JAMA Netw Open.* 2022;5(1):e2141995. doi:10.1001/jamanetworkopen.2021.41995

eTable 1. Estimated Latent Profile Analysis for Past-Year Prescription Drug Misuse

eTable 2. Estimated Latent Profile Analysis for Past-Year Prescription Opioid Misuse

eTable 3. Estimated Latent Profile Analysis for Past-Year Prescription Stimulant Misuse

eTable 4. Estimated Latent Profile Analysis for Past-Year Prescription Sedative or Tranquilizer Misuse

eTable 5. Multinomial Logistic Regression Assessing Characteristics Associated With Membership in Prescription Opioid Misuse Trajectories

eTable 6. Multinomial Logistic Regression Assessing Characteristics Associated With Membership in Prescription Stimulant Misuse Trajectories

eTable 7. Multinomial Logistic Regression Assessing Characteristics Associated With Membership in Prescription Sedative or Tranquilizer Misuse Trajectories

This supplemental material has been provided by the authors to give readers additional information about their work.

© 2022 McCabe SE et al. *JAMA Network Open.*

eTable 1. Estimated Latent Profile Analysis for Past-Year Prescription Drug Misuse

Estimated LPA means for the five class solution (Mplus Results)	18	19/20	21/22	23/24	25/26	27/28	29/30	35	40	45	50
No Misuse Trajectory (n = 10129)	0.000	0.000	0.000	0.000	0.000	0.000	0.000	0.000	0.000	0.000	0.000
Age 18 Peak Trajectory (n = 9194)	1.480	.608	.657	.310	.257	.138	.142	.069	.073	.115	.258
Ages 19/20 Peak Trajectory (n = 1621)	3.388	4.686	2.164	.565	.478	.205	.207	.085	.069	.124	.203
Ages 23/24 Peak Trajectory (n = 978)	2.295	2.345	3.131	4.541	1.691	.423	.438	.138	.131	.168	.455
Ages 27/28 Peak Trajectory (n = 594)	2.611	2.504	2.777	2.540	2.600	4.311	1.948	.365	.285	.485	.708
Age 35 Peak Trajectory (n = 373)	2.067	1.699	1.821	1.334	1.829	1.308	1.540	4.18	.563	.932	.909
Age 40 Peak Trajectory (n = 343)	1.865	1.527	1.297	1.060	.760	.567	.781	.714	4.38	1.49	1.45
Age 45 Peak Trajectory (n = 341)	1.655	1.195	1.038	.678	.546	.293	.455	.288	.255	4.76	.272
High risk Trajectory	3.185	3.286	3.848	3.586	3.572	3.832	3.668	4.673	4.939	3.342	3.181

© 2022 McCabe SE et al. *JAMA Network Open.*

(n = 254)											
**Classes**	Entropy	BIC	AIC	VLMR[a]	Sig.	LRT[b]	Sig.				
**1**	--	398828.614	398732.978	--	--	--	--				
**2**	0.933	378559.006	378411.204	20345.773	p<.001	20169.314	p<.001				
**3**	0.902	369322.615	369122.648	9312.556	p<.001	9312.556	p<.001				
**4**	0.881	363384.229	363132.097	5447.663	p<.001	5400.415	p<.001				
**5**	0.886	358116.439	357812.142	4419.312	p<.001	4380.984	p<.001				
**6**	0.882	352765.168	352408.706	5075.377	p<.001	5031.358	p<.001				
**7**	0.879	349023.753	348615.126	3391.352	p=.016	3361.939	p=.016				
**8**	0.861	345524.471	345063.679	2898.984	p<.001	2873.841	p<.001				
**9**	0.863	342591.394	342078.437	3076.316	p=.393	3049.636	p=.395				
**10**	0.879	340061.837	339496.714	2730.787	p=.328	2707.103	p=.331				

Footnote: Data source was Monitoring the Future study, 1976-1986 cohorts. Analyses do not use attrition weights.
[a]Vuong-Lo-Mendell-Rubin Likelihood Ratio Test.
[b]Lo-Mendell-Rubin Adjusted LRT Test.

© 2022 McCabe SE et al. *JAMA Network Open.*

eTable 2. Estimated Latent Profile Analysis for Past-Year Prescription Opioid Misuse

Estimated LPA means for the five class solution (Mplus Results)	18	19/20	21/22	23/24	25/26	27/28	29/30	35	40	45	50
**No Misuse Trajectory (n = 18366)**	0.000	0.000	0.000	0.000	0.000	0.000	0.000	0.000	0.000	0.000	0.000
**Age 18 Peak Trajectory (n = 4617)**	.904	.292	.324	.259	.210	.091	.127	.146	.124	.357	.405
**Ages 19/20 Peak Trajectory (n = 331)**	1.493	3.892	1.322	.628	.322	.116	.195	.197	.095	.314	.403
**Ages 27/28 Peak Trajectory (n = 230)**	.821	.803	1.610	1.380	1.785	3.727	1.620	.843	.700	1.187	1.295
**Age 40 Peak Trajectory (n = 257)**	.537	.324	.350	.346	.369	.233	.659	1.524	4.320	2.468	1.861
**Classes**	Entropy	BIC	AIC	VLMR[a]	Sig.	LRT[b]	Sig.				
**1**	--	129235.879	129160.574	--	--	--	--				
**2**	0.959	123360.317	123243.938	5940.637	p<.001	5883.629	p<.001				
**3**	0.916	119759.244	119601.789	3666.149	p<.001	3630.968	p<.001				
**4**	0.911	117173.151	116974.622	2651.167	p<.001	2625.726	p<.001				
**5**	0.889	115373.652	115134.047	1733.709	p=.357	1717.072	p=.362				
**6**	0.909	113132.837	112852.157	1818.426	p=.632	1800.976	p=.634				

Footnote: Data source was Monitoring the Future study, 1976-1986 cohorts. Analyses do not use attrition weights.
[a]Vuong-Lo-Mendell-Rubin Likelihood Ratio Test.
[b]Lo-Mendell-Rubin Adjusted LRT Test.

© 2022 McCabe SE et al. *JAMA Network Open.*

eTable 3. Estimated Latent Profile Analysis for Past-Year Prescription Stimulant Misuse

Estimated LPA means for the five class solution (Mplus Results)	**18**	**19/20**	**21/22**	**23/24**	**25/26**	**27/28**	**29/30**	**35**	**40**	**45**	**50**
**No Misuse Trajectory (n = 12602)**	0.000	0.000	0.000	0.000	0.000	0.000	0.000	0.000	0.000	0.000	0.000
**Age 18 Peak Trajectory (n = 8022)**	1.618	.604	.811	.520	.154	.138	.102	.061	.014	.012	.016
**Ages 19/20 Peak Trajectory (n = 2063)**	3.052	4.555	2.280	1.133	.245	.252	.174	.086	.018	.010	.011
**Ages 25/26 Peak Trajectory (n = 760)**	2.230	2.502	2.917	3.045	4.360	1.863	1.023	.367	.060	.033	.023
**Age 40 Peak Trajectory (n = 132)**	2.333	2.196	2.206	1.852	1.475	1.703	1.451	2.772	5.129	1.048	.186
**Age 45 Peak Trajectory (n = 133)**	1.928	1.867	2.176	1.666	.929	1.873	2.482	.534	.051	4.803	.206
**Age 50 Peak Trajectory (n = 89)**	2.136	1.307	1.759	1.207	.914	.961	.792	.910	1.100	1.492	4.803
**Classes**	Entropy	BIC	AIC	VLMR[a]	Sig.	LRT[b]	Sig.				
**1**	--	284835.344	284744.139	--	--	--	--				
**2**	0.959	267525.702	267384.749	17383.390	p<.001	17229.395	p<.001				
**3**	0.939	256574.667	256383.964	11040.019	p<.001	10942.218	p<.001				
**4**	0.948	248142.008	247901.557	10072.549	p=.014	9983.319	p=.015				
**5**	0.890	245274.736	244984.536	4364.173	p<.001	4325.511	p<.001				
**6**	0.896	240137.114	239797.166	3550.142	p<.001	3518.692	p<.001				
**7**	0.924	233973.668	233583.972	3665.586	p=.223	3633.114	p=.226				
**8**	0.930	231811.721	231372.276	-12.042	p=.746	-11.936	p=.746				

Footnote: Data source was Monitoring the Future study, 1976-1986 cohorts. Analyses do not use attrition weights.
[a]Vuong-Lo-Mendell-Rubin Likelihood Ratio Test.
[b]Lo-Mendell-Rubin Adjusted LRT Test.

© 2022 McCabe SE et al. *JAMA Network Open.*

eTable 4. Estimated Latent Profile Analysis for Past-Year Prescription Sedative or Tranquilizer Misuse

**Estimated LPA means for the five class solution (Mplus Results)**	**18**	**19/20**	**21/22**	**23/24**	**25/26**	**27/28**	**29/30**	**35**	**40**	**45**	**50**
**No Misuse Trajectory (n = 15596)**	0.000	0.000	0.000	0.000	0.000	0.000	0.000	0.000	0.000	0.000	0.000
**Age 18 Peak Trajectory (n = 6631)**	1.016	.328	.373	.309	.255	.152	.170	.177	.191	.133	.391
**Ages 19/20 Peak Trajectory (n = 720)**	2.376	3.74	1.595	.908	.535	.278	.422	.316	.230	.083	.301
**Ages 27/28 Peak Trajectory (n = 491)**	1.767	1.438	2.277	2.262	2.599	3.778	2.196	1.471	1.038	.528	1.030
**Age 45 Peak Trajectory (n = 381)**	.734	.419	.642	.595	.727	.539	.718	1.154	1.611	4.558	1.869
**Classes**	Entropy	BIC	AIC	VLMR[a]	Sig.	LRT[b]	Sig.				
**1**	--	212907.415	212823.004	--	--	--	--				
**2**	0.952	201002.638	200872.184	11974.819	p<.001	11865.136	p<.001				
**3**	0.908	196112.564	195936.068	4960.117	p<.001	4914.684	p<.001				
**4**	0.896	193088.887	192866.348	3093.720	p<.001	3065.383	p<.001				
**5**	0.892	190356.542	190087.961	3124.161	p=.695	3095.545	p=.695				
**6**	0.894	187581.168	187266.544	2478.701	p=.187	2455.998	p=.191				

Footnote: Data source was Monitoring the Future study, 1976-1986 cohorts. Analyses do not use attrition weights.
[a]Vuong-Lo-Mendell-Rubin Likelihood Ratio Test.
[b]Lo-Mendell-Rubin Adjusted LRT Test.

© 2022 McCabe SE et al. *JAMA Network Open.*

eTable 5. Multinomial Logistic Regression Assessing Characteristics Associated With Membership in PrescriptionOpioid Misuse Trajectories

	Age 18 Peak Trajectory	Age 19/20 Peak Trajectory	Age 27/28 Peak Trajectory	Age 40 Peak Trajectory
**Baseline Variables**	AOR(95% CI)	AOR(95% CI)	AOR(95% CI)	AOR(95% CI)
	n = 19,901	n = 19,901	n = 19,901	n = 19,901
**Sex**				
**Male**	Reference	Reference	Reference	Reference
**Female**	.941(.860,1.03)	.806(.612,1.06)	.849(.613,1.17)	.951(.677,1.33)
**Race/Ethnicity**				
**White**	Reference	Reference	Reference	Reference
**Black**	.450(.359,.563)	.305(.121,.771)	.126(.031,.514)	.357(.134,.952)
**Hispanic**	.685(.509,.923)	.612(.209,1.78)	.362(.107,1.22)	.811(.281,2.34)
**Other**	.690(.564,.845)	1.08(.616,1.91)	.692(.314,1.52)	.534(.215,1.32)
**Parental education**				
**Less than a BA**	Reference	Reference	Reference	Reference
**BA or higher**	1.21(1.10,1.33)	1.63(1.22,2.17)	1.00(.696,1.45)	.857(.590,1.24)
**GPA**				
**B- or higher**	Reference	Reference	Reference	Reference
**C+ or lower**	1.00(.906,1.11)	.883(.631,1.23)	1.07(.769,1.51)	.849(.563,1.28)
**College plans**				
**No**	Reference	Reference	Reference	Reference
**Yes**	.960(.871,1.05)	.780(.573,1.06)	.886(.620,1.26)	.898(.629,1.28)
**Region**				
**Northeast**	Reference	Reference	Reference	Reference
**Midwest**	1.03(.917,1.15)	.903(.605,1.34)	1.22(.743,2.03)	.782(.495,1.23)
**South**	1.03(.909,1.16)	1.05(.699,1.60)	1.28(.768,2.14)	.703(.428,1.15)
**West**	1.53(1.33,1.75)	.979(.632,1.51)	1.62(.966,2.73)	1.58(.950,2.65)
**Urbanicity**				
**Urban**	Reference	Reference	Reference	Reference
**Suburban**	1.00(.901,1.12)	.861(.600,1.23)	1.48(.939,2.33)	1.48(.985,2.24)
**Rural**	.882(.780,.996)	1.19(.805,1.76)	1.38(.843,2.28)	1.39(.876,2.21)
**Cohort year**				
**1976-1978**	Reference	Reference	Reference	Reference
**1979-1981**	.919(.820,1.02)	.842(.596,1.19)	.598(.401,.893)	1.30(.434,.867)
**1982-1984**	.938(.834,1.05)	.805(.558,1.16)	.657(.423,1.01)	2.28(.559,1.11)
**1985-1986**	.979(.858,1.11)	.619(.392,.977)	.681(.415,1.11)	3.59(.614,1.33)
**Cigarette Use**				

© 2022 McCabe SE et al. *JAMA Network Open.*

**No**	Reference	Reference	Reference	Reference
**Yes**	1.42(1.29,1.57)	1.99(1.44,2.77)	1.61(1.10,2.35)	1.68(1.10,2.58)
**Binge drinking**				
**No**	Reference	Reference	Reference	Reference
**Yes**	1.37(1.23,1.57)	1.73(1.19,2.52)	1.19(.794,1.78)	1.97(1.27,3.05)
**Marijuana**				
**No**	Reference	Reference	Reference	Reference
**Yes**	2.79(2.51,3.09)	4.39(2.79,6.88)	3.39(2.17,5.27)	1.47(.981,2.20)
**Lifetime Medical & NonmedicalUse (age 18) – Opioids[a]**				
**Never**	Reference	Reference	Reference	Reference
**Medical Only**	1.57(1.15,2.14)	5.42(1.56,18.7)	2.79(.733,10.6)	.997(.345,2.88)
**Nonmedical Only**	8.45(5.84,12.2)	15.5(4.33,55.8)	5.41(1.91,15.3)	.808(.254,2.57)
**Both**	10.4(7.48,14.5)	14.6(4.93,43.5)	5.74(2.21,14.9)	1.02(.324,3.21)

Notes: *p<.05, **p<.01, ***p<.001. All estimates provided use weights to adjust for attrition at age 50.
[a]Separate analyses with lifetime medical and nonmedical use at age 18 could only include one-sixth of the sample due to form specific questions given to respondents at baseline (n = 3,533).

© 2022 McCabe SE et al. *JAMA Network Open.*

eTable 6. Multinomial Logistic Regression Assessing Characteristics Associated With Membership in Prescription Stimulant Misuse Trajectories

	Age 18 Peak Trajectory	Ages 19/20 Peak Trajectory	Age 25/26 Peak Trajectory	Age 40 Peak Trajectory	Age 45 Peak Trajectory	Ages 50 Peak Trajectory
**Baseline Variables**	AOR(95% CI)	AOR(95% CI)	AOR(95% CI)	AOR(95% CI)	AOR(95% CI)	AOR(95% CI)
	n = 19,900	n = 19,900	n = 19,900	n = 19,900	n = 19,900	n = 19,900
**Sex**						
**Male**	Reference	Reference	Reference	Reference	Reference	Reference
**Female**	1.12(1.04,1.21)	1.27(1.12,1.44)	1.15(.942,1.41)	1.14(.753,1.74)	1.05(.650,1.69)	1.34 (.800,2.26)
**Race/Ethnicity**						
**White**	Reference	Reference	Reference	Reference	Reference	Reference
**Black**	.545(.465,.640)	.309(.210,.455)	.355(.191,.658)	.233(.066,.824)	.361(.082,1.57)	.526(.159,.1.74)
**Hispanic**	.733(.575,.934)	.881(.574,1.35)	.795(.414,1.52)	.109(.015,.786)	1.56(.548,4.44)	.304(.042,2.20)
**Other**	.905(.763,1.07)	1.00(.719,1.39)	1.13(.729,1.76)	.135(.033,.554)	.751(.247,2.28)	1.01(.290,3.52)
**Parental education**						
**Less than a BA**	Reference	Reference	Reference	Reference	Reference	Reference
**BA or higher**	1.03(.954,1.11)	1.08(.951,1.24)	.879(.706,1.09)	.738(.462,1.18)	.593(.313,1.12)	.853(.474,1.53)
**GPA**						
**B- or higher**	Reference	Reference	Reference	Reference	Reference	Reference
**C+ or lower**	1.08(.993,1.18)	.913(.794,1.05)	1.10(.889,1.37)	1.11(.674,1.83)	1.01(.608,1.70)	1.50(.858,2.62)
**College plans**						
**No**	Reference	Reference	Reference	Reference	Reference	Reference
**Yes**	.989(.911,1.07)	.979(.856,1.11)	.827(.662,1.03)	.530(.315,.891)	1.05(.673,1.64)	.770(.408,1.45)
**Region**						
**Northeast**	Reference	Reference	Reference	Reference	Reference	Reference
**Midwest**	1.07(.973,1.18)	1.27(1.08,1.48)	1.81(1.38,2.37)	1.45(.777,2.73)	2.56(1.08,6.02)	1.47(.738,2.96)
**South**	1.06(.955,1.18)	1.10(.927,1.32)	1.55(1.16,2.09)	1.77(.902,3.47)	2.15(.794,5.83)	1.67(.793,3.54)
**West**	1.05(.934,1.19)	1.26(1.03,1.55)	1.89(1.37,2.61)	2.74(1.36,5.52)	6.77(2.83,16.2)	2.30(.941,4.38)
**Urbanicity**						
**Urban**	Reference	Reference	Reference	Reference	Reference	Reference
**Suburban**	1.08(.988,1.19)	.957(.835,1.13)	1.33(1.03,1.72)	1.55(.854,2.83)	1.15(.597,2.22)	1.62(.828,3.20)
**Rural**	1.08(.982,1.20)	1.11(.949,1.31)	1.15(.881,1.51)	1.20(.645,2.23)	.935(.485,1.80)	1.41(.673,2.96)
**Cohort year**						
**1976-1978**	Reference	Reference	Reference	Reference	Reference	Reference
**1979-1981**	1.10(.997,1.21)	1.35(1.16,1.56)	.460(.369,.574)	.847(.495,1.44)	.904(.505,1.61)	.851(.400,1.80)

© 2022 McCabe SE et al. *JAMA Network Open.*

**1982-1984**	.965(.873,1.06)	.766(.650,.902)	.272(.206,.358)	1.14(.655,1.99)	.455(.233,.888)	1.03(.501,2.12)
**1985-1986**	.738(.658,.827)	.416(.323,.536)	.112(.071,.177)	.985(.521,1.86)	.380(1.74,.828)	1.35(.660,2.79)
**Cigarette smoking**						
**No**	Reference	Reference	Reference	Reference	Reference	Reference
**Yes**	1.44(1.32,1.58)	1.74(1.51,2.01)	1.88(1.50,2.37)	.899(.574,1.40)	1.01(.616,1.68)	1.14(.667,1.96)
**Binge drinking**						
**No**	Reference	Reference	Reference	Reference	Reference	Reference
**Yes**	1.61(1.48,1.76)	2.10(1.80,2.44)	1.47(1.15,1.88)	1.22(.732,2.05)	4.05(2.09,7.86)	2.21(1.10,4.40)
**Marijuana use**						
**No**	Reference	Reference	Reference	Reference	Reference	Reference
**Yes**	2.64(2.42,2.89)	4.39(3.68,5.23)	2.64(2.04,3.41)	3.11(1.83,5.27)	1.02(.590,1.76)	1.60(.832,3.08)
**Lifetime Medical & Nonmedical Use (age 18) – Stimulants[a]**						
**Never**	Reference	Reference	Reference	Reference	Reference	Reference
**Medical Only**	1.51(1.01,2.25)	1.40(.567,3.49)	.450(.093,2.15)	-- (--,--)[b]	-- (--,--)[b]	-- (--,--)[b]
**Nonmedical Only**	8.13(6.34,10.4)	4.79(2.88,7.97)	2.13(1.07,4.26)	4.65(1.35,16.0)	1.56(.734,3.31)	2.21(.450,10.8)
**Both**	6.93(4.85,9.91)	3.82(2.13,6.84)	1.55(.655,3.68)	6.20(1.39,27.6)	.623(.083,4.67)	2.82(.369,21.5)

Notes: *p<.05, **p<.01, ***p<.001. All estimates provided use weights to adjust for attrition at age 50.
[a]Separate analyses with lifetime medical and nonmedical use at age 18 could only include one-sixth of the sample due to form specific questions given to respondents at baseline (n = 3,600).
[b] '--' indicates that no respondents who were in this trajectory group indicated medical only use at baseline.

© 2022 McCabe SE et al. *JAMA Network Open.*

eTable 7. Multinomial Logistic Regression Assessing Characteristics Associated With Membership in Prescription Sedative/Tranquilizer Misuse Trajectories				
	**Age 18 Peak Trajectory**	**Ages 19/20 Peak Trajectory**	**Ages 27/28 Peak Trajectory**	**Age 45 Peak Trajectory**
**Baseline Variables**	AOR(95% CI)	AOR(95% CI)	AOR(95% CI)	AOR(95% CI)
	n = 19,908	n = 19,908	n = 19,908	n = 19,908
**Sex**				
**Male**	Reference	Reference	Reference	Reference
**Female**	1.16(1.07,1.24)	1.02 (.836,1.26)	1.02(.797,1.30)	1.14(.866,1.50)
**Race/Ethnicity**				
**White**	Reference	Reference	Reference	Reference
**Black**	.426(.386,.554)	.450(.168,.709)	.350(.165,.740)	.372(.189,.730)
**Hispanic**	.751(.585,.963)	.861(.473,1.56)	.773(.365,1.63)	1.08(.490,2.41)
**Other**	.787(.659,.940)	.929(.595,1.45)	1.24(.739,2.10)	.904(.460,1.77)
**Parental education**				
**Less than a BA**	Reference	Reference	Reference	Reference
**BA or higher**	1.15(1.06,1.25)	1.07(.868,1.32)	.812(.625,1.05)	.970(.721,1.30)
**GPA**				
**B- or higher**	Reference	Reference	Reference	Reference
**C+ or lower**	1.06(.969,1.16)	.829(.660,1.04)	1.33(1.04,1.69)	1.05(.765,1.46)
**College plans**				
**No**	Reference	Reference	Reference	Reference
**Yes**	.975(.895,1.06)	.915(.741,1.12)	.990(.765,1.28)	1.11(.827,1.49)
**Region**				
**Northeast**	Reference	Reference	Reference	Reference
**Midwest**	.864(.778,.960)	.869(.662,1.14)	.846(.605,1.18)	.847(.591,1.21)
**South**	1.23(1.11,1.38)	1.54(1.17,2.03)	1.32(.954,1.83)	1.20(.830,1.74)
**West**	1.22(1.08,1.39)	.741(.528,1.03)	.696(.442,1.09)	1.09(.713,1.68)
**Urbanicity**				
**Urban**	Reference	Reference	Reference	Reference
**Suburban**	1.18(1.07,1.30)	.896(.688,1.16)	1.06(.762,1.47)	1.09(.756,1.58)
**Rural**	1.12(1.01,1.25)	1.14(.863,1.51)	1.27(.921,1.77)	.815(.553,1.20)
**Cohort year**				
**1976-1978**	Reference	Reference	Reference	Reference
**1979-1981**	.827(.748,.914)	.634(.497,.810)	.474(.358,.629)	1.52(1.04,2.20)
**1982-1984**	.765(.689,.849)	.459(.352,.598)	.509(.373,.693)	1.51(1.03,2.21)
**1985-1986**	.678(.603,.763)	.414(.299,.574)	.523(.356,.769)	1.52(1.00,2.34)

© 2022 McCabe SE et al. *JAMA Network Open.*

**Cigarette smoking**				
**No**	Reference	Reference	Reference	Reference
**Yes**	1.52(1.39,1.66)	1.36(1.09,1.71)	1.73(1.29,2.33)	1.40(1.03,1.90)
**Binge drinking**				
**No**	Reference	Reference	Reference	Reference
**Yes**	1.28(1.17,1.40)	1.95(1.53,2.49)	1.58(1.18,2.12)	1.54(1.10,2.15)
**Marijuana use**				
**No**	Reference	Reference	Reference	Reference
**Yes**	2.35(2.14,2.58)	4.74(3.68,6.11)	2.88(2.09,3.99)	1.56(1.12,2.18)
**Lifetime Medical & Nonmedical Use (age 18) - Tranquilizers or Sedatives[a]**				
**Never**	Reference	Reference	Reference	Reference
**Medical Only**	1.63(1.20,2.22)	1.75(.468,6.59)	1.91(.665,5.50)	1.37(.595,3.15)
**Nonmedical Only**	8.16(6.19,10.7)	10.4(5.08,21.4)	4.03(1.74,9.30)	1.51(.640,3.59)
**Both**	6.89(5.07,9.35)	10.9(5.09,23.6)	4.39(1.99,9.66)	1.41(.598,3.33)

Notes: *p<.05, **p<.01, ***p<.001. All estimates provided use weights to adjust for attrition at age 50.

[a]Separate analyses with lifetime medical and nonmedical use at age 18 could only include one-sixth of the sample due to form specific questions given torespondents at baseline (n = 3,630).

© 2022 McCabe SE et al. *JAMA Network Open.*
